# Pharmaceutical stability of compounded acetylcholine chloride intraocular solution for intracoronary provocative vasospasm testing

**DOI:** 10.3389/fcvm.2026.1802503

**Published:** 2026-06-03

**Authors:** Killian J. McCarthy, Ryan Greenhalgh, Erica MacDonald, Laura Cedro, Duane Pinto, Margaret Stephan, Eric A. Osborn

**Affiliations:** 1 Division of Cardiovascular Medicine, Beth Israel Deaconess Medical Center, Harvard Medical School, Boston, MA, United States; 2 Department of Pharmacy, Beth Israel Deaconess Medical Center, Harvard Medical School, Boston, MA, United States

**Keywords:** acetylcholine chloride, coronary function testing, epicardial spasm, microvascular spasm, pharmaceutical stability

## Abstract

**Introduction:**

Provocative coronary vasospasm testing requires intracoronary acetylcholine (ACh) administration, however an ACh drug formulation approved for intracoronary use is not commercially available. Invasive coronary vasomotor testing programs have thus resorted to reconstituting and diluting ophthalmic ACh solutions for off-label and off-target intracoronary use, despite a lack of comprehensive ACh stability testing data required by pharmacy compounding regulations USP <797 > . This study aims to characterize the stability of compounded ACh intraocular solutions according to current pharmacy guidelines for use in provocative intracoronary ACh vasospasm testing.

**Methods:**

Potency and stability testing was performed by an FDA and DEAS registered, A2LA ISO 17025:2017 accredited laboratory. Using a simple, 7-step compounding method, a single vial of intraocular ACh solution was reconstituted and diluted with 500 mL of dextrose 5% water (D5W) to a final concentration of 18.2 mcg/mL. Compounded ACh was then stored in 20 mL syringes at ambient room temperature (25 ± 2 °C and 60 ± 5% relative humidity) under normal fluorescent lighting to simulate routine cardiac catheterization laboratory conditions. The compounded ACh solution appearance, pH, particulate matter, concentration and potency were evaluated in triplicate at 0, 4, and 12 h after compounding.

**Results:**

The compounded ACh solution was clear and colorless with a pH of 4.2 across all time points. Subvisible particulate matter, assessed by light obscuration techniques, demonstrated that the quantity of >10 *μ*m and >25 *μ*m particles present in each syringe remained safely within USP <788 > specifications. Finally, using high-performance liquid chromatography – mass spectrometry, the compounded ACh solutions revealed no significant degradation during the studied time frame, with 96.7 ± 0.3% of the initial ACh retained at 12 h, satisfying USP <797 > regulations to maintain 90.0% - 110.0% of label claim (18.2 mcg/mL).

**Conclusion:**

Utilizing a novel compounding method, intraocular ACh reconstituted and diluted in D5W met all USP <797 > pharmacy stability regulatory criteria over 12 h under ambient room temperature storage conditions. This study thus establishes a simplified ACh compounding approach for invasive coronary function testing centers to generate an intracoronary ACh drug formulation for use within 12 h in provocative coronary vasospasm testing procedures.

## Introduction

Relationships between epicardial coronary stenosis, myocardial ischemia, and exercise-induced angina are well established, however up to half of patients that demonstrate signs and symptoms of myocardial ischemia will not have evidence of obstructive epicardial coronary artery disease (CAD) at angiography (1, 2). Furthermore, nearly 15% of patients admitted with acute coronary syndromes are without obstructive CAD (3–5). Patient outcomes associated with these clinical scenarios are not benign, often leading to poor quality of life, anti-anginal polypharmacy, and frequent medical contacts for refractory symptoms, including the potential for recurrent hospitalizations (6–8). Chronic angina without a defined cause thus often culminates in repeat non-invasive testing and invasive coronary angiography procedures, placing patients at unnecessary risks while also contributing to a high economic burden (9). Numerous studies implicate coronary vasomotor and structural microvascular abnormalities as the underlying mechanisms responsible for continued symptoms in patients without obstructive epicardial coronary disease (10–12). As such, comprehensive invasive coronary function testing (CFT) approaches that can simultaneously diagnose and guide treatment decisions tailored to specific coronary dysfunction endotypes holds significant potential to improve outcomes in this underserved population (13–16).

The clinical utility of performing comprehensive CFT assessment in patients experiencing persistent angina without obstructive epicardial coronary artery disease has recently gained traction (17), with both US and European cardiovascular society guidelines endorsing invasive guidewire-based hyperemic measurement of coronary flow reserve (CFR) and/or microcirculatory resistance plus intracoronary provocative coronary vasospasm testing with acetylcholine chloride (ACh) (17, 18). Despite supportive guidelines and the development of sensitive wire-based invasive physiology systems that can accurately interrogate coronary function, the creation of invasive CFT programs within US and European institutions has been limited in part due to the absence of a commercially available intracoronary ACh drug formulation approved for provocative coronary vasospasm testing (19). To overcome this barrier, CFT programs have thus resorted to reconstituting and diluting an ophthalmic ACh preparation for use as an intracoronary drug, typically used within 3 h of compounding (20).

While clinical studies demonstrate efficacy of the diluted ophthalmic ACh preparation to induce coronary vasospasm with a relatively low risk of adverse events (20, 21), there is a lack of available information regarding the potency and stability of the compounded ACh solution to support its use within the coronary arteries. As pharmacy compounding regulations USP <797 > require comprehensive stability data to prepare off-label drugs in this manner and establish an effective “beyond use date” (BUD) that defines the acceptable timeframe for clinical use of the compounded formulation, the absence of published stability data on the diluted ophthalmic ACh solution represents safety and operational barriers for intracoronary ACh use at invasive CFT centers. The aim of this study is to comprehensively assess the stability of compounded ACh intraocular solutions for intracoronary use in line with current pharmacy compounding guidelines, in order to facilitate provocative ACh testing for coronary vasospasm and provide a pathway for new CFT centers to secure pharmacy approval.

## Materials and methods

This single-center, collaborative study between the cardiology division and pharmacy departments at Beth Israel Deaconess Medical Center, Harvard Medical School (Boston MA) assessed the pharmaceutical potency and stability of diluted ACh chloride intraocular solution for provocative testing in the assessment of coronary vasospasm. Potency and stability testing was performed in an FDA and DEA registered, A2LA ISO 17025:2017 accredited laboratory (ARL Bio Pharma, Oklahoma City OK) providing analytical and microbiological testing services. Prior to sample testing, a stability indicating method (proprietary to ARL Bio Pharma) was first developed and validated per USP <1225 > for the quantitation of ACh, which includes accuracy, linearity, precision, range, specificity, and system specificity. Specificity demonstrates non-interference from impurities and matrix components and involves stress studies/forced degradation to confirm the method can separate degradation products from ACh, the active pharmaceutical ingredient (API).

### Sample preparation

**Figure 1:**
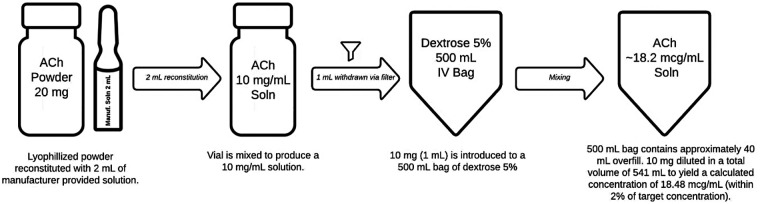
A novel 7-step compounding method to simplify intracoronary ACh solution preparation for use in provocative coronary vasospasm testing.

A simple 7-step compounding method was created to simplify intracoronary ACh solution preparation off-site by pharmacy teams or at point-of-care within the cardiac catheterization laboratory (Figure 1). Miochol^™^-E intraocular solution (Bausch & Lomb, Germany; NDC 24208–539–20), a drug indicated for use to constrict the pupil during eye surgery, is commercially packaged as a 20 mg vial of ACh chloride lyophilized sterile powder with a 2 mL electrolyte diluent ampule composed of sodium acetate trihydrate, potassium chloride, magnesium chloride hexahydrate, calcium chloride dihydrate and sterile water for injection. Under aseptic conditions, the individual components included in the Miochol^™^-E package were reconstituted per manufacturer protocol to create a 10 mg/mL working solution of Ach chloride. The 10 mg/mL ACh solution was next filtered with a manufacturer provided 0.22 µm disk filter, and 1 mL (10 mg) of the solution injected into a 500 mL bag of 5% dextrose injection (Baxter Healthcare Cooperation, Deerfield IL; NDC 0338–0017–03). A labeled 500 mL bag for intravenous administration will contain excess solution from the primary manufacturer (referred to as overfill) to maintain the labeled volume over the course of the product shelf-life. The assigned overfill amount from manufacturers of 500 mL bags is estimated to be 40 mL on average. This means the aliquot prepared (10 mg ACh in a total volume of 541 mL) yields a calculated ACh solution concentration of 18.48 mcg/mL. Next, 20 mL of the diluted ACh solution was drawn into a 30 mL polypropylene Luer-Lok syringe (Becton Dickinson and Company, Franklin Lakes NJ; product code 302832) and sealed with a tamper evidence syringe cap (International Medical Industries, Pompano Beach FL; product code 57–25-CE). For the purposes of the current study, due to the short timeline of scheduled testing points, ACh solution sample preparation and dilution was performed in-house by an FDA and DEAS registered, A2LA ISO 17025:2017 accredited testing laboratory (ARL Bio Pharma). After preparation, syringes containing compounded ACh solutions were stored at ambient room temperature (25 ± 2 °C and 60 ± 5% relative humidity) under normal fluorescent lighting. A total of eighty-four containers were prepared for comprehensive ACh stability testing at 0, 4, and 12 h following compounding as detailed below. Microbiological stability (sterility and endotoxin testing) was not assessed due to the product's relatively short beyond-use date being assigned, and the environment the compounded product was produced in being a cleanroom suite. The risk to product sterility is more associated with the environment the compound is produced in, the sterility of the individual components, and the products final container (which did undergo container-closure integrity testing) rather than the specific formulation, which is only using sterile components. The environment these are compounded in meets the standards of USP <797 > category 2 sterile compounding, which have maximum allowable beyond-use dates of 10 days when stored under refrigeration and 4 days when stored at controlled room temperature. If the intention for this specific formulation was to assign beyond-use dates longer than the category-2 maximum beyond-use dating, sterility and endotoxin testing would be warranted.

### Physical evaluation

Physical stability of compounded ACh chloride solutions were assessed by visual inspection. Samples were evaluated against black and white backgrounds for visible particulate matter, cloudiness/turbidity, and changes in color. Container closure integrity was performed a 0 and 12 h after compounding via vacuum decay. pH samples were taken at each time point in accordance with USP <791 > specifications. Sub-visible particulate testing was performed via light obscuration particle count testing in accordance with USP <788> Method 1 testing protocols.

### Potency assessment

High-performance liquid chromatographic – tandem mass spectrometry (LC-MS) analysis was utilized for ACh chloride concentration or potency evaluation using the previously validated, stability indicating method (*n* = 3 samples per timepoint analyzed). Analyses were performed on a UHPLC system (Thermo Ultimate 3000 or Vanquish; Thermo Fisher Scientific, Waltham MA) equipped with a temperature-controlled autosampler and column compartment. Chromatographic separation was achieved on an Ethylene Bridged Hybrid Amide XP column (3.0 mm x 50 mm; 2.5 µm, 130 Å; Waters XBridge, Milford MA) maintained at 40 °C. The mobile phase consisted of 15 mM ammonium formate (pH 3.0, adjusted with formic acid) and acetonitrile in a 15:85 (v/v) ratio, delivered isocratically at 0.5 mL/min. The autosampler was maintained at 5 °C to preserve sample integrity, and the injection volume was 10 µL. Total run time was 3 min per injection. Detection was carried out on a Thermo Quantiva or TSQ Vantage triple-stage quadrupole mass spectrometer (Thermo Fisher Scientific) operated in positive-ion electrospray mode. Multiple reaction monitoring transitions were monitored at mass to charge ratio (m/z) 146.1 → 87.1 for ACh and m/z 155.1 → 87.1 for the internal standard, ACh-d₉ chloride (Sigma Aldrich, St Louis MO). Collision energy was set at 14 V for both transitions, with the following source settings: spray voltage 4.00 kV, sheath gas 50 arbitrary units, auxiliary gas 15 arbitrary units, ion transfer tube temperature 350 °C, and vaporizer temperature 400 °C. Calibration standards at five levels (70%, 85%, 100%, 115%, and 130% of target concentration) were prepared daily from freshly made stock solutions and contained a fixed internal standard concentration. Calibration curves were constructed by plotting analyte/internal standard peak area ratios against nominal concentrations using linear regression. The injection sequence included system equilibration, blanks, calibration standards, samples, and bracketing standards (100% level) after no more than 30 sample injections. System suitability criteria included calibration curve linearity (R^2^ ≥ 0.99), precision (%RSD ≤5%), response factor consistency (95%–105%) when calculated against the calibration curve, and carryover control (blank ≤1% of prior standard), assessed before and after sample analysis. Sample concentration quantification was performed using Chromeleon software (Thermo Fisher Scientific). Measured concentrations were multiplied by the known sample dilution factor to determine final concentration assay values, and are reported as mean ± standard deviation.

## Results

A stability indicating method per USP <1225 > was developed and validated for the quantitation of ACh, which included accuracy, linearity, precision, range, specificity, and system specificity. ACh chloride intraocular solutions (18.2 mcg/mL) in D5W were prepared using a new, simplified 7-step compounding method to reduce potential dilutional errors and improve efficient clinical workflow, and then tested using the validated stability indicating method over a 12-hour time period.

The compounded ACh solution remained clear and colorless at 0 and 12 h, while maintaining a stable pH (4.2) throughout the 12-hour testing period. Container closure integrity was uncompromised at both 0 and 12 h, with no detected leaks by vacuum decay interrogation. In accordance with USP <788> Method 1 testing, acceptable passing sub-visible particle number size limits were defined as ≤6000 particles/container for ≥10 µm particle size and ≤600 particles/container for ≥25 µm particle size. For the ≥10 µm size particles, testing demonstrated that at 0 h there were 966 particles detected, decreasing sequentially over time to 342 particles at 4 h and 182 particles at 12 h, thus satisfying the defined passing criteria. For the ≥25 µm size particles, only 4 particles were identified at 0 h, followed by 0 and 2 particles detected at 4 and 12 h, respectively, similarly meeting USP <788 > passing criteria.

**Figure 2:**
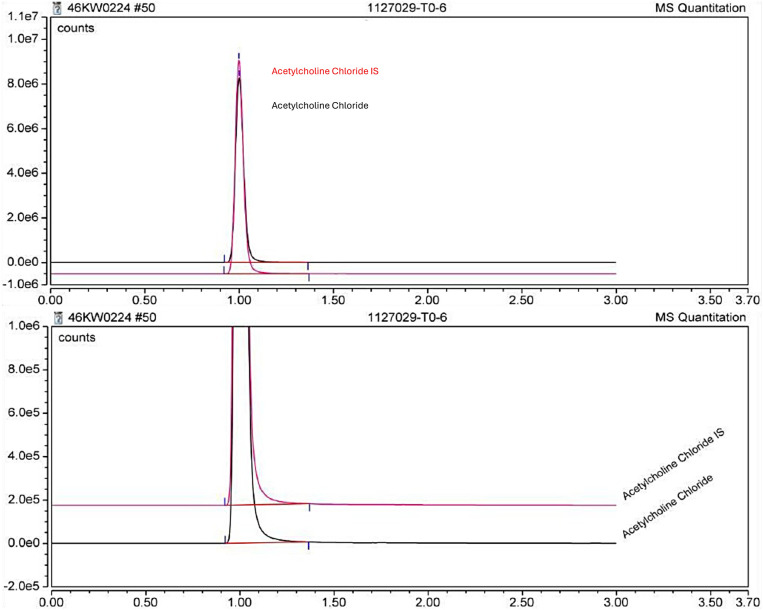
Acetylcholine chloride and internal standard reinjection at time 0**,**Lot 1127029 T0-6 sample (18.1846 mcg/mL) - signal intensity vs. retention time (eluted at approximately 1 min).

**Figure 3:**
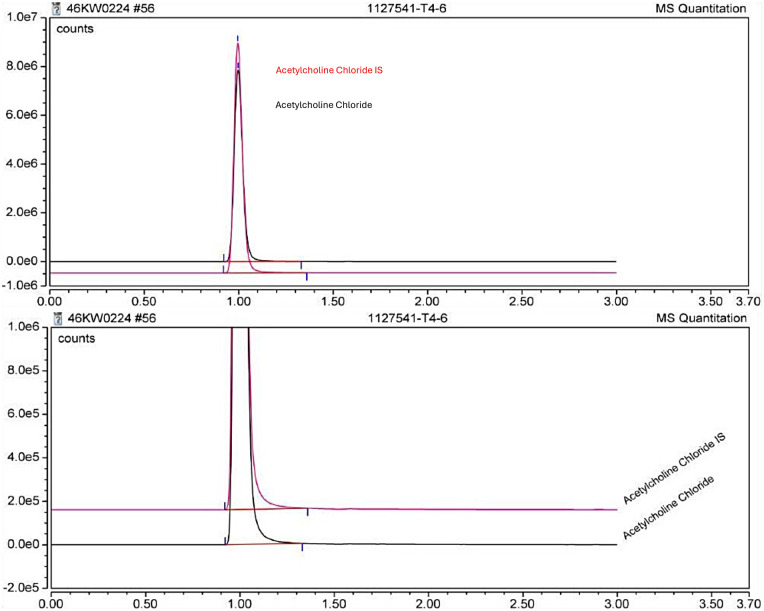
Acetylcholine chloride and internal standard reinjection at 4 h, Lot 1127541 T4-6 sample (17.5378 mcg/mL) - signal intensity vs. retention time (eluted at approximately 1 min).

**Figure 4:**
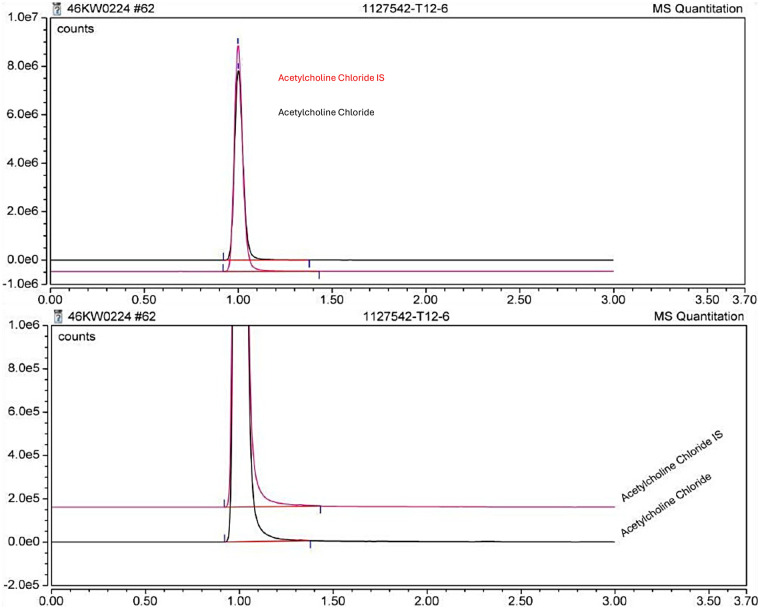
Acetylcholine chloride and internal standard reinjection at 12 h, Lot 127542 T12-6 sample (17.6254 mcg/mL) - signal intensity vs. retention time (eluted at approximately 1 min).

Next, potency testing of the compounded ACh solutions at 0, 4, and 12 h was performed using LC-MS analysis as specified in detail above. According to USP <797 > pharmacy compounding guidelines and official monographs of USP-National Formulary approved products, in order for potency testing to meet passing criteria and support specific BUD labeling, the compounded ACh concentration measured at each timepoint is required to reside within 90%–110% of the concentration label claim. Specifically, for the purposes of the present study, in order to satisfy conditions for BUD labeling the final ACh solution concentration at 0, 4, and 12 h after compounding must remain between 16.4 and 20.0 mcg/mL. Immediately after compounding at 0 h, the baseline ACh concentration relative to the expected 18.2 mcg/mL starting reference concentration was 99.9 ± 0.3%. Subsequently, the relative ACh concentration was measured as 96.1 ± 0.4% at 4 h and 96.7 ± 0.3% at 12 h, thus remaining within accepted USP <797> BUD labeling specifications. A comprehensive summary of the complete ACh stability study testing results is presented in Table 1. Chromatic peaks of ACh chloride vs. ACh-d9 chloride internal standard reinjected samples are presented in Figures 2–4. All RSD meet acceptance criteria of <5%. In fact, across multiple replicate sets, the RSD of measured concentrations was consistently below 1%, with a representative RSD of approximately 0.4%. This indicates an analytical variability of approximately ±1% under the conditions used in this study. A list of samples tested via LC-MS is presented in Table 2.

**Table 1:** Results of comprehensive stability testing for intracoronary ACh chloride solutions (18.2 mcg/mL) stored 20 mL in 30 mL polypropylene syringes at ambient room temperature.

Attribute	Methods	Specification	Timepoints		
			Initial (0 h)	4 h	12 h
Appearance	Visual Examination	Clear, colorless solution	Conforms	N/A	Conforms
pH	USP <791>	N/A	4.2	4.2	4.2
Subvisible Particulate Matter	USP <788> Method 1	≥ 10 µm; ≤ 6,000 particles/container	966	342	182
		≥ 25 µm; ≤ 600 particles/container	4	0	2
Container Closure Integrity	Vacuum Decay	No leaks detected	Conforms	N/A	Conforms
Relative ACh Chloride Concentration (Potency)	Liquid Chromatography-Mass Spectrometry	90.0% - 110% of Label Claim (18.2 mcg/mL)	99.9 ± 0.3%	96.1 ± 0.4%	96.7 ± 0.3%
Average ACh Chloride Concentration (*n* = 6 runs each)	Liquid Chromatography-Mass Spectrometry	18.2 mcg/mL Label Claim	18.2 ± 0.1 mcg/mL	17.5 ± 0.1 mcg/mL	17.6 ± 0.1 mcg/mL

**Table 2:** Chromatographic performance and quantitative results for ACh chloride stability samples by high-performance liquid chromatography – tandem mass spectrometry.

#	Injection name	Retention time (min) Acetylcholine chloride	Retention time (min) Acetylcholine chloride IS	Area Counts*min Acetylcholine chloride	Area Counts*min Acetylcholine chloride IS	Area Ratio Acetylcholine Chloride	Amount Acetylcholine Chloride
1	Discard 00 chk	1	0.99	61	13	4.7643	0.1001
2	Discard 00 chk	1.01	1.01	2,298	755,775	0.003	0.0001
3	Discard cal chk	1.01	1.01	328,633	556,287	0.5908	0.0124
4	Discard cal chk	1.01	1	385,495	534,356	0.7214	0.0152
5	Discard cal chk	1.01	1	440,484	520,444	0.8464	0.0178
6	Discard cal chk	1.01	1	494,534	513,561	0.963	0.0202
7	Discard cal chk	1.01	1	555,441	505,603	1.0986	0.0231
8	Discard 00 chk	0.7	0.7	71	61	1.1519	0.0242
9	Discard 00 chk	1.01	N/A	41	N/A	N/A	N/A
10	Discard 0 chk	1	1	2,066	690,077	0.003	0.0001
11	00	1	0.7	40	57	0.7096	0.0149
12	0	1.01	1	2,032	681,749	0.003	0.0001
13	70%	1	1	304,380	522,244	0.5828	0.0123
14	70%	1	1	304,604	519,972	0.5858	0.0123
15	70%	1	1	305,636	520,371	0.5873	0.0124
16	70%	1	1	305,512	523,723	0.5833	0.0123
17	70%	1	1	304,467	522,284	0.583	0.0123
18	70%–6	1	1	303,984	517,575	0.5873	0.0124
19	85%	1	1	362,141	511,349	0.7082	0.0149
20	85%	1	1	364,227	511,831	0.7116	0.015
21	85%	1	1	364,174	512,571	0.7105	0.0149
22	85%	1	1	364,456	511,053	0.7131	0.015
23	85%	1	1	363,538	508,588	0.7148	0.015
24	85%–6	1	1	362,882	508,212	0.714	0.015
25	100%	1	1	421,285	503,456	0.8368	0.0176
26	100%	1	1	419,556	504,721	0.8313	0.0175
27	100%	1	1	422,876	500,813	0.8444	0.0178
28	100%	1	1	420,746	502,499	0.8373	0.0176
29	100%	1	1	422,600	505,648	0.8358	0.0176
30	100%–6	1	1	420,672	501,914	0.8381	0.0176
31	115%	1	1	478,986	496,305	0.9651	0.0203
32	115%	1	1	480,621	496,542	0.9679	0.0204
33	115%	1	1	476,941	493,773	0.9659	0.0203
34	115%	1	1	476,775	492,895	0.9673	0.0203
35	115%	1	1	477,115	494,086	0.9657	0.0203
36	115%–6	1	1	478,116	494,818	0.9662	0.0203
37	130%	1	1	537,470	492,054	1.0923	0.023
38	130%	1	1	533,603	491,386	1.0859	0.0228
39	130%	1	1	534,214	492,089	1.0856	0.0228
40	130%	1	1	536,822	494,012	1.0867	0.0228
41	130%	1	1	535,504	492,009	1.0884	0.0229
42	130%–6	1	1	532,190	491,401	1.083	0.0228
43	0	1	1	1,901	631,922	0.003	0.0001
44	00	0.99	0.99	51	88	0.5796	0.0122
45	1127029-T0	1	1	426,190	493,312	0.8639	18.1699
46	1127029-T0	1	1	426,085	492,520	0.8651	18.1946
47	1127029-T0	1	1	425,178	492,029	0.8641	18.174
48	1127029-T0	1	1	426,676	491,595	0.8679	18.254
49	1127029-T0	1	1	424,121	493,610	0.8592	18.0709
50	1127029-T0-6	1	1	425,195	491,762	0.8646	18.1846
51	1127541-T4	1	0.99	418,629	504,261	0.8302	17.4612
52	1127541-T4	1	0.99	415,704	503,630	0.8254	17.3611
53	1127541-T4	1	0.99	418,702	500,725	0.8362	17.5873
54	1127541-T4	1	0.99	417,155	501,297	0.8322	17.5025
55	1127541-T4	1	0.99	414,666	499,346	0.8304	17.4662
56	1127541-T4-6	1	0.99	416,239	499,188	0.8338	17.5378
57	1127542-T12	1	1	412,176	495,510	0.8318	17.4956
58	1127542-T12	1	1	412,658	494,025	0.8353	17.5686
59	1127542-T12	1	1	412,294	491,778	0.8384	17.6331
60	1127542-T12	1	1	414,149	493,947	0.8384	17.6347
61	1127542-T12	1	1	412,893	492,793	0.8379	17.6224
62	1127542-T12-6	1	1	413,977	494,003	0.838	17.6254
63	100% Bracket	1	1	414,693	493,281	0.8407	0.0177
64	100% Bracket	1	1	412,823	491,360	0.8402	0.0177
65	100% Bracket	1	1	415,193	491,618	0.8445	0.0178
66	100% Bracket	1	1	414,041	490,704	0.8438	0.0177
67	100% Bracket	1	1	411,835	492,851	0.8356	0.0176
68	100% Bracket-6	1	1	412,111	493,772	0.8346	0.0176

ACh and the internal standard (ACh-d9 chloride) were consistently detected with retention times of approximately 1 min across all analytical runs. No significant retention time drift was observed between calibration standards, quality control samples, or stability samples. Calibration standards demonstrated a monotonic increase in peak area ratios with increasing analyte concentration. The internal standard response remained consistent across all calibration levels, with no evidence of ion suppression. Zero blank and double blank samples (“0” and “00”) exhibited minimal analyte response with area ratios near 0.003 and calculated amounts at or near the lower limit of quantitation, confirming the absence of significant carryover following high-concentration injections. Bracketed 100% calibration checks showed reproducible area ratios (0.8356–0.8445) and calculated amounts consistent with nominal values, confirming acceptable suitability and calibration stability throughout the analytical sequence.

Six replicate injection of the T0 samples (Sample ID: 1127029-T0) demonstrated consistent analyte response. Calculated ACh chloride amounts ranged from 18.1 to 18.3 mcg/mL, with minimal variability across injections. The reinjected T0 sample (Figure 2) showed comparable results, indicating no degradation during the short-term sample handling or autosampler residence. Samples analyzed at the T4 time point (Figure 3, Sample ID: 1127541-T4) produced calculated acetylcholine chloride amounts ranging from 17.4 to 17.6 mcg/mL across replicate injections. Relative to T0, the measured concentration showed a modest decrease, consistent across all replicates. T12 stability samples (Figure 4, Sample ID: 1127542-T12) demonstrated calculated amounts ranging from 17.5 to 17.6, with excellent agreement among replicates. The reinjected sample (T12–6) produced a value of 17.6, further confirming analyte stability through the longest evaluated time point. Acetylcholine chloride demonstrated consistent chromatographic behavior and reproducible quantitative response by LC-MS across all evaluated time points. Measured concentrations at T4 and T12 remained closely aligned with T0 values, with no evidence of significant degradation or analytical instability.

## Discussion

ACh is a carboxylic ester that is a naturally occurring neurohormone which mediates nerve impulse transmission throughout the autonomic nervous system. After release from the pre-synaptic nerve ending, it is rapidly inactivated within seconds *in vivo* through hydrolysis by the enzyme acetylcholinesterase to acetic acid and choline. Direct application of ophthalmic Michol^TM^-E ACh chloride solution to the eye surface causes ACh-mediated smooth muscle relaxation leading to rapid and short acting miosis, that has been used successfully for decades to facilitate eye surgical procedures. When administered within arterial beds, intracoronary ACh results in several physiologic responses that: 1) initiate endothelium-dependent relaxation of the smooth muscle cells located in the medial artery layer via a nitric oxide pathway, and 2) activate the contractile process of smooth muscle cells via increased calcium influx (22, 23). With normal endothelial function, nitric oxide mediated smooth muscle relaxation is the predominant response to ACh exposure, leading to coronary artery vasodilation. However, in patients with underlying endothelial dysfunction, the healthy coronary vasodilatory nitric oxide response mechanisms are impaired, resulting in more pronounced direct smooth muscle vasoconstriction that manifests as epicardial and/or microvascular spasm. In this way, intracoronary ACh administration functions as an ideal exogenous provocation agent for use in the cardiac catheterization laboratory when assessing for clinical coronary artery vasospasm (24).

Our study investigated the pharmaceutical stability of compounded ACh 18.2 mcg/mL intraocular solution in 5% dextrose for use in provocative coronary artery vasospasm testing. While ACh chloride is generally acknowledged as unstable *in vivo* due to rapid degradation through hydrolysis by acetylcholinesterase it is previously known that ester molecules such as ACh also have the potential to be degraded *ex vivo* via hydrolysis reactions without the presence of acetylcholinesterase, such as under routine aqueous storage conditions as tested in the current study (25–27). Factors that diminish the rate of non-enzymatic ACh hydrolysis include acidic pH and lower temperature, which both promote greater ACh stability. Despite intrinsic hydrolysis degradation, the results of our study demonstrate that compounded ACh chloride in 5% dextrose solutions maintained physical and chemical stability over a 12-hour period after compounding is performed. Thus, using our new, simplified 7-step method therefore allows off-site pharmacy personnel or catheterization lab staff at the point-of-care broad latitude to prepare a single batch of compounded ACh solutions for intracoronary use that can then be deployed intraprocedurally throughout the duration of standard daytime hours when invasive CFT procedures are typically performed. During testing, each test dose of ACh (1 mL, 2.5 mL, 5 mL, +/- 10 mL) is removed from the single Luer-Lok syringe of 30 mL of ACh solution and diluted to 10 mL final total volume with 5% dextrose in a single 10 mL Luer-Lok syringe for intracoronary administration over 30–60 s. Furthermore, our findings are especially pertinent to institutions actively conducting or newly considering initiating invasive CFT programs, by providing important information to help secure compounded intracoronary ACh pharmacy approval.

Examining our data further reveals that, across all timepoints over 12-hours, the ACh solution retained a clear, colorless appearance, exhibiting no discernible particulate formation beyond the accepted USP specified particle size limits. Compounded ACh solution pH remained consistently stable at 4.2 throughout the study period, a particularly noteworthy finding considering that ACh hydrolysis demonstrates a strong pH dependence with acidic conditions decelerating the hydrolysis rate (28). As per the manufacturer's specifications, the pH range for commercial Michol^TM^-E ACh chloride is 5.0–8.2, likely due to the sensitivity associated with the labeled indication and ophthalmic route of administration to ensure patient comfort. However, for intracoronary ACh administration, maintaining a lower pH as demonstrated in our testing results may confer beneficial conditions that increase ACh stability following compounding observed during the 12 h investigated in our work. Finally, container closure integrity testing revealed the absence of leaks over 12 h, thereby supporting the stability of polypropylene Luer-Lok syringes as a suitable storage container. This finding is significant as it provides strong evidence for the utilization of pre-drawn syringes of ACh chloride in the cardiac catheterization laboratory, offering opportunities to enhance clinical workflows and minimize procedural delays that can occur when waiting for compounded ACh syringe transport from remote pharmacy locations.

The crux of our data relies on the stability of compounded ACh solutions to retain suitable concentrations over time compared to that measured initially immediately after pharmacy compounding was performed. As discussed above, ACh chemical potency remained within the USP-acceptable range (90%–110% of label concentration claim) for all timepoints, with measured relative concentrations of 99.9 ± 0.3%, 96.1 ± 0.4%, and 96.7 ± 0.3% at 0, 4, and 12 h after ACh compounding, respectively. While slight decreases were observed in ACh concentrations compared to the initial time point, the magnitude of change was relatively small and consistent with the expected first-order degradation kinetics previously described by Wolfenden and Yang for ACh in aqueous media (29). This prior work extrapolated a first-order rate constant for uncatalyzed (or neutral) hydrolysis of ACh in water to be 3.9 × 10^−7^ s^−1^ at 25 °C (*Δ*H^‡^ = 20.0 kcal/mol; T*Δ*S^‡^ = −6.1 kcal/mol), which calculates to an estimated shelf life of approximately 3 days, assuming the degradation pathway in fact follows first-order kinetics. Notably, our results did not demonstrate a rapid or clinically significant decline in concentration during the 12-hour study period. These findings align with published degradation data suggesting that ACh chloride is most susceptible to degradation in an alkaline pH range and at elevated temperatures, neither condition of which were present in our 7-step intracoronary ACh compounding formulation (i.e., pH 4.2 at 25 °C) (29). Conversely, acidic, low-ionic strength environments, such as the dextrose solution we utilized as the diluent in our study, would be expected to provide enhanced ACh stability. Original studies utilized dextrose solution for dilution, which is why this diluent was chosen for testing. 0.9% sodium chloride could potentially be a more stable diluent, given it has a pH range of 4.5–7. With that said, without additional testing, we cannot say with certainty which diluent would be considered more “stable”.

Importantly, assignment of a 12-hour BUD allows for preparation to be performed within the pharmacy department ahead of scheduled cases. The assignment of a beyond use date to a compounded sterile preparation is determined on the compound's stability. Preparation of ACh solution in clinical environments such as the catheterization laboratory is more susceptible to microbial contamination from the external environment due to a lack of engineering controls, and there is also an increased risk for a compounding error. Manufacturers do not provide compounding instructions for this off-label usage and dilution, leaving institutions to develop their own internal procedures for compounding. Previous ACh stability studies performed have shown deviations of up to 230% from the target concentration, suggesting an error occurred during ACh compounding preparation (20). Simplification of the ACh compounding process as described in the new 7-step method utilized in our study, and routinely performing ACh compounding in the pharmacy department, are both expected to substantially reduce the risk of a compounding error to occur.

### Limitations

Several limitations to our study should be acknowledged. This study only examined compounded ACh solution stability over a 12-hour period at room temperature, and thus longer-term stability beyond 12 h under different storage conditions remains unknown. Despite this, it may be reasonably expected that compounded ACh stored with standard refrigeration (4 °C) may further decrease the potential for ACh degradation by reducing hydrolysis rates further, leading to an even longer shelf life. Only one concentration (18.2 mcg/mL) and one diluent (5% dextrose solution) were tested, making it uncertain whether our results would be different using alternative diluents such as normal saline. Sterility and endotoxin testing was not necessary to be performed in this study, primarily due to the assigned BUD being below USP <797 > category 2 compounded sterile preparation maximum allowable BUD, and 12 h at controlled room temperature being the maximum for category 1 compounded sterile preparations. Future stability studies could address these limitations by examining extended BUD durations, longer duration storage and/or under different environmental conditions, comparative stability across different concentrations and diluents, and inclusion of sterility/endotoxin testing.

## Conclusion

Utilizing a novel, simple 7-step compounding method, our comprehensive pharmaceutical stability testing results demonstrate that reconstituted and diluted intraocular ACh solutions (18.2 mcg/mL) in a 5% dextrose solution adhere to USP <797 > pharmacy compounding physical and chemical specifications for appearance, particulate matter, pH, container integrity, and potency for up to 12 h when stored at room temperature. Our results address an important barrier to pharmacy approval at CFT testing centers performing provocative invasive ACh coronary vasospasm testing procedures in the cardiac catheterization lab.

## Data Availability

The original contributions presented in the study are included in the article/Supplementary Material, further inquiries can be directed to the corresponding author.
